# Glutathione S-transferase omega genes in Alzheimer and Parkinson disease risk, age-at-diagnosis and brain gene expression: an association study with mechanistic implications

**DOI:** 10.1186/1750-1326-7-13

**Published:** 2012-04-11

**Authors:** Mariet Allen, Fanggeng Zou, High Seng Chai, Curtis S Younkin, Richard Miles, Asha A Nair, Julia E Crook, V Shane Pankratz, Minerva M Carrasquillo, Christopher N Rowley, Thuy Nguyen, Li Ma, Kimberly G Malphrus, Gina Bisceglio, Alexandra I Ortolaza, Ryan Palusak, Sumit Middha, Sooraj Maharjan, Constantin Georgescu, Debra Schultz, Fariborz Rakhshan, Christopher P Kolbert, Jin Jen, Sigrid B Sando, Jan O Aasly, Maria Barcikowska, Ryan J Uitti, Zbigniew K Wszolek, Owen A Ross, Ronald C Petersen, Neill R Graff-Radford, Dennis W Dickson, Steven G Younkin, Nilüfer Ertekin-Taner

**Affiliations:** 1 Mayo Clinic Florida, Department of Neuroscience, Jacksonville, FL, USA; 2 Mayo Clinic Minnesota, Department of Biostatistics, Rochester, MN, USA; 3 Mayo Clinic Florida, Biostatistics Unit, Jacksonville, FL, USA; 4 Mayo Clinic Minnesota, Gene Expression Core, Advanced Genome Technology Center, Rochester, MN, USA; 5 Department of Neurology, St.Olav's Hospital, Edvard Griegs Gate 8, 7006 Trondheim, Norway; 6 Department of Neuroscience, Norwegian University of Science and Technology, NTNU, 7491 Trondheim, Norway; 7 Department of Neurodegenerative Disorders, Medical Research Centre, Polish Academy of Sciences, Warsaw, Poland; 8 Mayo Clinic Florida, Department of Neurology, Jacksonville, FL, USA; 9 Mayo Clinic Minnesota, Department of Neurology, Rochester, MN, USA

**Keywords:** GSTO genes, Disease risk, Gene expression, Association

## Abstract

**Background:**

Glutathione S-transferase omega-1 and 2 genes (*GSTO1*, *GSTO2*), residing within an Alzheimer and Parkinson disease (AD and PD) linkage region, have diverse functions including mitigation of oxidative stress and may underlie the pathophysiology of both diseases. *GSTO *polymorphisms were previously reported to associate with risk and age-at-onset of these diseases, although inconsistent follow-up study designs make interpretation of results difficult. We assessed two previously reported SNPs, *GSTO1 *rs4925 and *GSTO2 *rs156697, in AD (3,493 ADs vs. 4,617 controls) and PD (678 PDs vs. 712 controls) for association with disease risk (case-controls), age-at-diagnosis (cases) and brain gene expression levels (autopsied subjects).

**Results:**

We found that rs156697 minor allele associates with significantly increased risk (odds ratio = 1.14, *p *= 0.038) in the older ADs with age-at-diagnosis > 80 years. The minor allele of *GSTO1 *rs4925 associates with decreased risk in familial PD (odds ratio = 0.78, *p *= 0.034). There was no other association with disease risk or age-at-diagnosis. The minor alleles of both *GSTO *SNPs associate with lower brain levels of *GSTO2 *(*p *= 4.7 × 10^-11^-1.9 × 10^-27^), but not *GSTO1*. Pathway analysis of significant genes in our brain expression GWAS, identified significant enrichment for glutathione metabolism genes (*p *= 0.003).

**Conclusion:**

These results suggest that *GSTO *locus variants may lower brain *GSTO2 *levels and consequently confer AD risk in older age. Other glutathione metabolism genes should be assessed for their effects on AD and other chronic, neurologic diseases.

## Background

Glutathione S-Transferase (GST) family of genes have been implicated in multiple neuropsychiatric [1-4] and neurodegenerative diseases [5-11]; where altered levels or function of these enzymes is thought to impact levels of oxidative stress and/or inflammation in a way that contributes to disease susceptibility. A linkage locus on chromosome 10q that has been implicated in both Alzheimer's (AD)[11-13] and Parkinson's disease (PD)[13] harbors two GST genes of the omega class: *GSTO1 *and *GSTO2*, which are approximately 75 kb apart.

GSTOs have enzymatic activities as thioltransferases and dehydroascorbate reductases that promote antioxidant activity and can also function in metabolism of drugs and toxins[14]. Additionally, *GSTO1 *was shown to promote activation of the pro-inflammatory cytokine, interleukin-1β (IL-1β) by post-translational processing[15]. Given their location and function, they have been studied as candidate genes in AD and PD[5,6,9,11,14,16-18]. Li et al. compared hippocampal gene expression levels in 6 AD vs. 2 control brains and identified significantly lower *GSTO1 *levels in the AD hippocampi[5]. This group studied AD and PD families that showed linkage to chromosome 10, using the age-at-onset phenotype [13] and identified association of multiple SNPs at the *GSTO *locus with delayed age-at-onset of both diseases[5], with the strongest effects observed for *GSTO1 *rs4925 and *GSTO2 *rs2297235 SNPs that are in tight linkage disequilibrium (LD). No significant influence was detected for either AD or PD risk in this study.

Since this initial report, several follow-up studies have been published with mixed approaches and results. Kölsch et al. reported association of rs4925 with earlier age-at-onset of AD, thus in opposite direction to the original report[6], and no effect on AD risk. Lee et al. found modest association of rs4925 with AD risk in Carribean-Hispanic families that show linkage to chromosome 10q[11] as did Capurso et al. in an Italian case-control series[9], though neither study detected an age-at-onset effect. A case-control study by Wahner et al. was the only report for an effect of *GSTO *locus on PD risk, with both rs4925 and rs2297235 conferring protection, especially in those with smoking history[16]. Additionally, several studies reported lack of association with age-at-onset or risk of AD[17,18] or PD[14].

Additional investigation of the *GSTO *locus is needed to further elucidate the role of these genetic variants in AD and PD, especially given the potential to establish the glutathione metabolism as a molecular pathway that is common to multiple, chronic neurologic diseases. An important shortcoming of most prior reports on the *GSTO *locus is the modest sample sizes, which could underlie the inconsistent results likely due to lack of power, sample or locus heterogeneity or a combination of these factors. Both AD[19,20] and PD[21] are complex diseases with substantial genetic component. Some of the genetic risk for these diseases has been identified via linkage and association studies and shown to influence age-at-onset[19-23]. More recently, genome-wide association studies (GWAS) of AD[24-28] and PD[29,30], with sample sizes exceeding 10,000 subjects provide considerably greater power for detection of susceptibility loci. Despite their advantages, GWAS do not explain all of the underlying genetic component of these and other complex diseases, thus necessitating alternative approaches[31], including analysis of quantitative phenotypes.

In this study, we assessed the *GSTO *locus for its role in AD and PD, using an in-depth approach aimed at surmounting these challenges. Given the original report of association with delayed age-at-onset of AD and PD [5,7], and with risk of AD in some follow-up studies [9,11], we postulate that *GSTO *locus variants confer risk of LOAD in older age. We have a collection of > 8,000 late-onset AD (LOAD) case-controls, which includes a large series of older subjects ≥ 80 years of age-at-diagnosis/death (clinical/autopsied LOADs) or evaluation (controls). We analyzed two previously reported, coding SNPs in *GSTO1 *and *GSTO2 *for association both with disease risk and age-at-diagnosis in the LOAD series, as well as a large PD series. Reduced expression levels of *GSTO1 *[5] and other glutathione metabolism genes [10,32] have been reported in AD. We therefore analyzed the *GSTO *SNPs for association with brain *GSTO1 *and *GSTO2 *levels in > 750 brain samples from autopsied subjects with AD and other brain pathologies to determine whether they influenced disease risk by affecting brain gene expression. In an expression GWAS (eGWAS) testing association of 24,526 transcript levels measured in these brain samples with 213,528 *cis*SNPs within ± 100 kb of the tested transcript, we identified 686 genes that have significant *cis*SNP/transcript associations (in-press, *PLoS Genetics*). We analyzed these genes to discover molecular pathways that are enriched for genes with significant brain *cis*SNPs, and identified glutathione metabolism as one of the top pathways. Our results suggest that *GSTO *locus variants influence brain *GSTO2 *levels and confer AD risk at older age. These findings have mechanistic implications for the *GSTO *locus and glutathione metabolism genes, which should be explored further in AD and other chronic, neurologic diseases to identify functional variants that influence disease risk by altering brain gene expression levels.

## Results

### Association of *GSTO *locus SNPs with LOAD and PD risk

**Figure 1:**
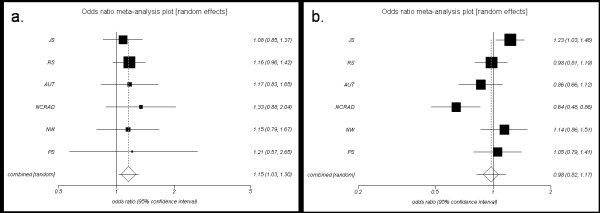
**Meta-analysis of rs156697 in LOAD: a) Older LOAD series with ages > 80 years; b) Younger LOAD series with ages between 60-80 years**. Combined series p value of association with LOAD risk is *p *= 0.018 in the older and *p *= 0.79 in the younger LOAD series. Breslow-Day test for series heterogeneity *p *value = 0.97 in the older and *p *= 0.004 in the younger series.

*GSTO *locus SNPs rs156697 and rs4925 were tested for association with disease risk in an older series (> 80 years) of 1,368 LOADs vs. 1,623 controls; in a younger series of 2,193 LOADs vs. 3,060 controls (60-80 years) and in 678 PDs vs. 712 controls (Table 1 Table 2 Table 3 and Table 4), using logistic regression analysis. *GSTO2 *rs156697 was significantly associated with LOAD in the older series (*p *= 0.038), with the minor allele conferring increased risk (OR = 1.14, 95% confidence interval = 95%CI = 1.01-1.30) (Table 5). There was no other significant disease risk association in the combined younger LOAD series (Table 6) or LOAD series of all ages (Table 7), although there were trends for increased LOAD risk with the minor allele of rs156697 in the LOAD series of all ages (*p *= 0.18, OR = 1.06) and of rs4925 in the older LOAD series (*p *= 0.15, OR = 1.10). Analysis of the six individual LOAD series of the older age group revealed consistently increased risk estimates for rs156697 (Table 5), which is also evident from the meta-analysis (Figure 1a), where there is no evidence of between-series heterogeneity (Breslow-Day *p *= 0.97). In contrast, the younger LOAD series is significantly heterogeneous (Breslow-Day *p *= 0.004), with three series showing increased (JS, NW, PS) and the other three (RS, AUT, NCRAD) with protective risk estimates (Table 6 Figure 1b).

**Table 1:** LOAD case-control series demographics: LOAD series over age 80

Diagnosis	Series	N	Mean Age (range)	Males (%)	ApoE4+ (%)
	ALL	1,368	84 (80-105)	437 (32)	722 (53)

	JS	315	84 (80-95)	114 (36)	171 (54)

	RS	306	86 (80-104)	113 (37)	127 (42)

AD cases	AUT	314	87 (80-105)	100 (32)	193 (61)

	NCRAD	153	84 (80-98)	45 (29)	93 (61)

	PS	101	83 (80-90)	24 (24)	45 (45)

	NW	179	86 (80-96)	41 (23)	93 (52)

	ALL	1,623	84 (80-100)	636 (39)	347 (21)

	JS	322	85 (80-100)	137 (43)	71 (22)

	RS	973	84 (80-99)	393 (40)	219 (23)

Controls	AUT	102	86 (80-98)	49 (48)	16 (16)

	NCRAD	86	87 (80-99)	32 (37)	10 (12)

	PS	22	85 (80-91)	5 (23)	3 (14)

	NW	118	85 (80-96)	20 (17)	28 (24)

**Table 2:** LOAD case-control series demographics: LOAD series ages 60-80

Diagnosis	Series	N	Mean Age (range)	Males (%)	ApoE4+ (%)
	ALL	2,193	74 (61-80)	872 (40)	1,539 (70)

	JS	549	74 (61-80)	212 (39)	378 (69)

	RS	291	74 (61-80)	122 (42)	201 (69)

AD cases	AUT	267	74 (61-80)	139 (52)	159 (60)

	NCRAD	542	73 (61-80)	199 (37)	451 (83)

	PS	378	75 (64-80)	137 (36)	226 (60)

	NW	166	74 (61-80)	63 (38)	124 (75)

	ALL	3,060	73 (60-80)	1,404 (46)	776 (25)

	JS	650	73 (60-80)	271 (42)	200 (31)

	RS	1,433	75 (60-80)	720 (50)	351 (24)

Controls	AUT	258	72 (61-80)	158 (61)	64 (25)

	NCRAD	122	72 (61-80)	48 (39)	24 (20)

	PS	164	72 (64-80)	38 (23)	33 (20)

	NW	433	73 (61-80)	169 (39)	104 (24)

**Table 3:** LOAD case-control series demographics: LOAD series All Ages

Diagnosis	Series	N	Mean Age (range)	Males (%)	ApoE4+ (%)
	ALL	3,561	78 (61-105)	1,309 (37)	2,261 (63)

	JS	864	78 (61-95)	326 (38)	549 (64)

	RS	597	80 (61-104)	235 (39)	328 (55)

AD cases	AUT	581	81 (61-105)	239 (41)	352 (61)

	NCRAD	695	75 (61-98)	244 (35)	544 (78)

	PS	479	77 (64-90)	161 (34)	271 (57)

	NW	345	80 (61-96)	104 (30)	217 (63)

	ALL	4,683	77 (60-100)	2,073 (44)	1,122 (24)

	JS	972	77 (60-100)	408 (42)	271 (28)

	RS	2,406	78 (60-99)	1,113 (46)	570 (24)

Controls	AUT	360	76 (61-98)	207 (58)	80 (22)

	NCRAD	208	78 (61-99)	80 (38)	34 (16)

	PS	186	73 (64-91)	43 (23)	36 (19)

	NW	551	75 (61-96)	222 (40)	132 (24)

**Table 4:** PD case-control series demographics

Diagnosis	Series	N	Mean Age (range)	Males (%)	ApoE4+ (%)
PD (Sporadic)		421	65 (25-94)	150 (36)	121 (29)

PD (Familial)	PD (USA)	257	62 (32-89)	93 (36)	71 (28)

PD (All)		678	64 (25-94)	243 (36)	192 (28)

Control		712	66 (18-89)	299 (42)	198 (28)

**Table 5:** Association of *GSTO *locus SNPs with LOAD risk in the older LOAD series with ages > 80 years

**N (MAF)**							
**rs#**	**Locus**	**Series**	**AD**	**Control**	**OR**	**95% CI**	**p-value**

		**All**	**1,338 (0.37)**	**1,604 (0.33)**	**1.14**	**1.01-1.30**	**0.038**

		JS	309 (0.36)	319 (0.34)	1.09	0.85-1.41	NS

		RS	299 (0.37)	963 (0.33)	1.21	0.98-1.48	0.073

rs156697	GSTO2	AUT	311 (0.40)	99 (0.36)	1.19	0.81-1.75	NS

		NCRAD	146 (0.37)	86 (0.30)	1.29	0.81-2.05	NS

		NW	176 (0.33)	116 (0.30)	1.19	0.81-1.76	NS

		PS	97 (0.38)	21 (0.33)	1.38	0.62-3.11	NS

		All	1,341 (0.33)	1,595 (0.30)	1.10	0.97-1.25	0.151

		JS	306 (0.31)	321 (0.30)	1.08	0.23-1.41	NS

		RS	302 (0.33)	960 (0.30)	1.17	0.95-1.44	0.134

rs4925	GSTO1	AUT	313 (0.35)	99 (0.35)	1.00	0.69-1.46	NS

		NCRAD	149 (0.34)	80 (0.28)	1.28	0.80-2.05	NS

		NW	173 (0.30)	114 (0.28)	1.13	0.76-1.70	NS

		PS	98 (0.35)	21 (0.33)	1.11	0.48-2.58	NS

Results of multivariate logistic regression analysis are shown for each SNP, each series individually and for the combined series. N = number of subjects, MAF = minor allele frequency, OR = odds ratio; NS = not significant.

**Table 6:** Association of *GSTO *locus SNPs with LOAD risk in the younger LOAD series with ages between 60-80 years

**N (MAF)**							
**rs#**	**Locus**	**Series**	**AD**	**Control**	**OR**	**95% CI**	**p-value**

		All	2,152 (0.35)	3,013 (0.34)	0.99	0.90-1.11	NS

		JS	544 (0.37)	634 (0.33)	1.24	1.03-1.49	**0.025**

		RS	287 (0.34)	1,423 (0.34)	0.99	0.80-1.21	NS

rs156697	GSTO2	AUT	263 (0.34)	254 (0.38)	0.88	0.67-1.15	NS

		NCRAD	531 (0.34)	119 (0.45)	0.58	0.41-0.82	**0.002**

		NW	165 (0.33)	428 (0.30)	1.01	0.75-1.37	NS

		PS	362 (0.37)	155 (0.36)	0.99	0.71-1.37	NS

		All	2,148 (0.32)	3,011 (0.31)	1.00	0.90-1.11	NS

		JS	544 (0.33)	644 (0.29)	1.16	0.96-1.40	0.119

		RS	289 (0.30)	1,414 (0.31)	0.95	0.77-1.17	NS

rs4925	GSTO1	AUT	265 (0.29)	254 (0.34)	0.85	0.64-1.12	0.248

		NCRAD	522 (0.31)	117 (0.31)	0.86	0.6-1.24	NS

		NW	158 (0.31)	423 (0.29)	0.94	0.69-1.29	NS

		PS	370 (0.35)	159 (0.34)	0.94	0.67-1.31	NS

Results of multivariate logistic regression analysis are shown for each SNP, each series individually and for the combined series. N = number of subjects, MAF = minor allele frequency, OR = odds ratio; NS = not significant.

**Table 7:** Association of *GSTO *locus SNPs with LOAD risk in the LOAD series all ages combined

**N (MAF)**							
**rs#**	**Locus**	**Series**	**AD**	**Control**	**OR**	**95% CI**	**p-value**

		All	3,490 (0.36)	4,617 (0.34)	1.06	0.98-1.14	0.177

		**JS**	**853 (0.37)**	**953 (0.33)**	**1.19**	**1.03-1.38**	**0.021**

		RS	586 (0.35)	2,386 (0.33)	1.07	0.93-1.23	NS

rs156697	GSTO2	AUT	574 (0.38)	353 (0.37)	0.98	0.79-1.22	NS

		NCRAD	677 (0.35)	205 (0.39)	0.78	0.60-1.02	0.069

		NW	341 (0.33)	544 (0.30)	1.10	0.87-1.39	NS

		PS	459 (0.37)	176 (0.35)	1.00	0.75-1.35	NS

		All	3,489 (0.32)	4,606 (0.31)	1.03	0.95-1.12	NS

		JS	850 (0.32)	965 (0.30)	1.12	0.96-1.31	0.137

		RS	591 (0.31)	2,374 (0.31)	1.04	0.90-1.21	NS

rs4925	GSTO1	AUT	578 (0.33)	353 (0.34)	0.89	0.71-1.11	NS

		NCRAD	671 (0.31)	197 (0.29)	1.01	0.76-1.34	NS

		NW	331 (0.31)	537 (0.29)	1.03	0.81-1.32	NS

		PS	468 (0.35)	180 (0.34)	0.93	0.69-1.26	NS

Results of multivariate logistic regression analysis are shown for each SNP, each series individually and for the combined series. N = number of subjects, MAF = minor allele frequency, OR = odds ratio; NS = not significant.

PD series were composed of those with (PD FAM) or without (PD-SPO) family history of PD (Table 4). Assessment of these individual series revealed significant association of rs4925 with lower PD risk in the familial PD series (*p *= 0.034, OR = 0.78) and a trend for decreased risk with rs156697 (*p *= 0.116, OR = 0.83) in this series (Table 8). There was no association with PD risk in the sporadic PD or the combined series.

**Table 8:** Association of *GSTO *locus SNPs with PD risk

**N (MAF)**							
**rs#**	**Locus**	**Series**	**PD**	**Control**	**OR**	**95% CI**	**p-value**

		PD-All	661 (0.35)	702 (0.36)	0.94	0.80-1.11	NS

rs156697	GSTO1	PD-SPO	411 (0.36)	702 (0.36)	1.03	0.85-1.24	NS

		PD FAM	250 (0.31)	702 (0.36)	0.83	0.66-1.05	0.116

		PD-All	667 (0.30)	707 (0.33)	0.92	0.78-1.09	NS

rs4925	GSTO1	PD-SPO	416 (0.33)	707 (0.33)	1.03	0.85-1.25	NS

		**PD FAM**	**251 (0.27)**	**707 (0.33)**	**0.78**	**0.61-0.98**	**0.034**

Results of multivariate logistic regression analysis are shown for each SNP, each series individually and for the combined series. N = number of subjects, MAF = minor allele frequency, OR = odds ratio; NS = not significant.

### Association of *GSTO *locus SNPs with AD and PD age-at-diagnosis

We employed age-at-diagnosis (clinical) or death (autopsy) as the surrogate quantitative variable for age-at-onset in our LOAD subjects (3,561 LOADs), who had an age range of 61-105 (mean age = 78, Table 1 Table 2 and Table 3). Analysis of rs156697 and rs4925 did not identify any significant association with age-at-diagnosis in the combined or individual LOAD series, although there was a trend for rs156697 for delayed age-at-diagnosis in the combined series (*p *= 0.098) (Additional File 1: Table S1). There was no significant age-at-diagnosis association when the older and younger LOADs were assessed separately (data not shown). The combined PD series had an age range of 25-94 (mean age = 64, Table 4). There was no significant association of either *GSTO *SNP with PD age-at-diagnosis (Additional File 1: Table S1).

**Figure 2:**
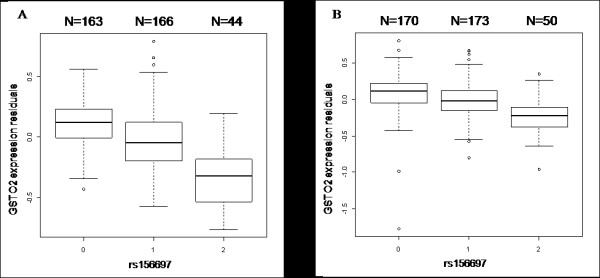
**Box plots of brain *GSTO2 *expression levels by rs156697 genotype: a**. Cerebellar measurements from combined autopsy series of 373 subjects (197 ADs, 176 controls) b. Temporal cortex measurements from combined autopsy series of 393 subjects (202 ADs, 191 controls). *GSTO2 *expression value residuals obtained after multivariate linear regression analysis are displayed in box plots according to the genotypes for rs156697. 0 = Homozygous Major (TT), 1 = Heterozygote (TC) and 2 = Homozygous Minor (CC). The number of subjects with each genotype is indicated above each box plot. The bottom and top of a box represent the lower and upper quartiles, respectively. The band near the middle of the box is the median. The ends of the whiskers depict the most extreme observations still within 1.5 inter quartile range of the corresponding quartile. Any data not included between the whiskers are plotted as dots.

### Association of *GSTO *locus SNPs with brain gene expression

In an eGWAS using 773 brain samples, we measured levels of 24,526 transcripts from the cerebellum and temporal cortex of autopsied subjects with and without AD pathology (in-press, *PLoS Genetics*). Control subjects without AD pathology often had other brain pathologies. We determined the association of rs156697 and rs4925 with brain *GSTO1 *and *GSTO2 *levels in these series, using linear regression analysis. Both SNPs had significant association with brain *GSTO2 *(Table 9, 10), but not with *GSTO1 *levels (data not shown). This association was significant for *GSTO2 *transcript levels measured from both the cerebellum and the temporal cortex; and in both the AD and control autopsy subjects, although the effect size estimates appeared to be bigger for the ADs and the cerebellum. The minor alleles of both SNPs were associated with lower brain *GSTO2 *levels in all analyses, with an additive pattern of association (Figure 2).

**Table 9:** Association of *GSTO *locus SNP rs156697 with brain *GSTO2 *expression levels

Tissue	Diagnosis	N	Beta	P-value
Cer	All	373	-0.200	1.90E-27
	
	AD	197	-0.225	1.12E-17
	
	Con	176	-0.173	1.15E-10

Tx	All	393	-0.146	1.20E-14
	
	AD	202	-0.166	2.50E-10
	
	Con	191	-0.119	3.30E-05

Results of multivariate linear regression analysis testing association of rs156697 with cerebellar (Cer) and temporal cortex (Tx) levels of *GSTO2 *in the autopsied subjects with AD pathology, those without (Con), and the combined (All) group. N = number of subjects. Beta coefficient and p value of association between the transcript levels and the SNP are shown for each analyzed series.

**Table 10:** Association of *GSTO *locus SNP rs4925 with brain *GSTO2 *expression levels

Tissue	Diagnosis	N	Beta	P-value
Cer	All	371	-0.175	6.88E-19
	
	AD	196	-0.203	2.23E-12
	
	Con	175	-0.149	4.91E-08

Tx	All	392	-0.132	4.70E-11
	
	AD	201	-0.167	3.93E-09
	
	Con	191	-0.089	2.30E-03

Results of multivariate linear regression analysis testing association of rs4925 with cerebellar (Cer) and temporal cortex (Tx) levels of *GSTO2 *in the autopsied subjects with AD pathology, those without (Con), and the combined (All) group. N = number of subjects. Beta coefficient and p value of association between the transcript levels and the SNP are shown for each analyzed series.

Twenty other *cis*SNPs at the *GSTO *locus were tested for association with brain *GSTO1 *and *GSTO2 *levels in our eGWAS. Although rs156697 had the strongest association, many of the additional *cis*SNPs also showed significant association with brain *GSTO2 *(Additional File 1: Table S2, Additional File 2: Figure S1), but not *GSTO1 *(data not shown) levels. The strongest *cis*SNPs were in an LD block encompassing *GSTO2 *(Additional File 2: Figure S1.)

### Discovery of glutathione metabolism pathway in a brain gene expression GWAS

In our brain eGWAS we identified 686 genes with cerebellar transcript levels that are significantly influenced by *cis*SNPs, which were submitted to pathway analysis[33] to discover molecular pathways that are significantly regulated in the brain. Glutathione metabolism was identified as one of the most significant pathways (*p *= 0.0035), where six genes from this pathway out of the thirty that existed within our eGWAS had significant *cis*SNPs that influence both the cerebellar and temporal cortex levels of these genes (Additional Files 3: Figure S2a and 4: Figure S2b). Five of the significant genes are enzymes that directly catalyze the binding of reduced glutathione to substrates (*GSTO2*, *GSTT1*, *GSTT2*, *GSTM3*, *GSTM5*) and *GCTG *is involved in amino acid metabolism, including glutamate (Additional File 1).

## Discussion

*GSTO1 *and *GSTO2*, which are evolutionarily conserved genes[14], previously implicated in AD[5,6,9,11] and PD[5,16], have diverse attributed functions including antioxidant activity via generation of ascorbate (Vitamin C) [14,34,35]; biotransformation of inorganic arsenic[14,34]; modulation of ryanodine receptors and thus calcium release and apoptosis[36]; and post-translational processing of the pro-inflammatory cytokine, IL-1β[15]. Given their functions which are relevant for the pathophysiology of neurodegenerative diseases and their location in linkage regions for AD[11-13] and PD[13], *GSTO *locus variants have previously been studied for their association with risk and age-at-onset of AD and PD with mixed results[5,6,9,11,14,16-18].

In this study, we assessed two coding polymorphisms, rs4925 (Ala140Asp) in *GSTO1 *and rs156697 (Asn142Asp) in *GSTO2 *in a large LOAD series of > 8,000 subjects, ~3,000 of whom were older (> 80 years) and in a PD series of > 1,300 subjects including both familial and sporadic cases. We found significant LOAD risk association for the minor allele of rs156697 in older subjects and a suggestive trend for delayed age-at-diagnosis. These results are consistent with the original[5] and some of the follow-up reports on this locus[9,11], and suggest that the reported delay in age-at-onset is likely to be due to an increased risk conferred in older subjects. Given the age-dependent decline in key glutathione metabolism components and their role in mitigating oxidative stress[32], the postulate that risky *GSTO *variants lead to increased risk in older LOADs due to accumulation of oxidative damage with increasing age, is a plausible scenario. It should be emphasized that our study utilized age-at-diagnosis as a surrogate for age-at-onset and unrelated case-controls, rather than family-based series. These differences could underlie the marginal age association in our study, in comparison to the original study[5].

Given the tight LD (r2 = 0.73, D' = 0.94 in HapMap3)[37] between the two coding SNPs tested for AD and PD risk association in this study, we did not correct for multiple testing. If corrected, the AD association in the older ADs would no longer be significant (*p *= 0.076). Furthermore, *GSTO *locus variants were not reported to have significant or suggestive association with AD risk in the recent, large GWAS[26-28]. Although, these findings could collectively suggest that the AD risk association in our study is a false positive, there are alternative explanations: First, the effect conferred by *GSTO2 *rs156697 is age-specific based on our results, and others[5]. Additionally, unlike the older series in our study, the younger LOAD series had significantly heterogeneous results for the rs156697 SNP. Thus, the large LOAD GWAS need to be re-analyzed focusing on the different age groups and also for age-at-onset or diagnosis association. Second, the effect of the *GSTO2 *variant is likely modest for LOAD risk, despite strong effects on brain gene expression. Third, although *GSTO2 *rs156697 has the strongest effect on brain expression of this gene in that locus, it may still not be the functional variant, thus leading to weak or heterogeneous effects on LOAD risk. Our results in LOAD risk and brain gene expression provide support for functional variant discovery efforts in the *GSTO2 *region and screening of such variants for their effects in transcriptional assays.

There was no significant association of *GSTO *SNPs with disease risk in the combined PD series. This may not be surprising given the difference in sample size and therefore power between the LOAD and PD series. Whereas our older LOAD series (1,338 LOAD vs. 1,604 controls) have ~61% power to detect the effect of the *GSTO2 *rs156697 SNP (OR = 1.14), the combined PD series (661 PDs vs. 702 controls) tested for this SNP, has ~32% power to detect this effect at α = 0.05. There was, however, association with decreased risk in the familial PD cases for the *GSTO1 *rs4925 minor allele. Although consistent with one other study in PD[16], this finding requires further replication. It is intriguing to note that this variant also conferred a protective effect in the LEAPS-PD GWAS, which assessed PD sib-pairs in its first stage[38,39]. The opposite direction of association in the familial PDs (and some of the younger LOAD series) vs. the older LOAD series could have several explanations including the tested SNPs not being functional themselves but marking different functional variants of opposing effects; heterogeneity due to different gene-gene or gene-environment interactions in different groups; and false positivity in some of the tested series.

Although both *GSTO *SNPs are in coding regions, they do not lead to any change in the enzymatic activities of *GSTO1*[34,35] or *GSTO2*[34]. While their effects on LOAD and PD could be due to other, untested alterations in protein function, another potential mechanism of action is influencing levels of gene expression. Indeed, both SNPs had highly significant effects on brain gene expression levels of *GSTO2*, but not *GSTO1*. Amongst the 22 *cis*SNPs tested for association with *GSTO *levels in our brain eGWAS, rs156697 had the strongest effect, where the risky minor allele was associated with lower brain *GSTO2 *levels. These results strongly suggest that the risk conferred by the *GSTO *locus is most likely due to variants which influence *GSTO2 *levels in the brain. These findings are biologically compatible with the very high antioxidant function of *GSTO2*, where its dehydroascorbate reductase activity was found to be 70-100% greater than that of *GSTO1*[34].

Brain expression levels of other key enzymes of glutathione metabolism are also significantly influenced by genetic variants, as was identified from pathway analysis of our significant brain eGWAS results. Given our findings with *GSTO2 *and other studies implicating glutathione metabolism genes in neurodegenerative diseases[8,10,32], it will be important to analyze these additional glutathione metabolism genes with high brain regulation, for variants that influence risk of AD and other neurodegenerative diseases.

In summary, our results support *GSTO2 *as a risk gene for older LOAD subjects, where risky genotypes reduce brain levels of this gene, which likely leads to accumulation of oxidative damage worsening with increasing age. These findings have implications for disease mechanism, as well as the search for genetic risk variants in AD and other neurodegenerative diseases. First, it will be important to analyze the existing large LOAD and PD risk GWAS by different age-strata and also using age-at-onset as the outcome, where available. Second, *GSTO2 *should be sequenced for variants that may influence gene expression and thereby disease risk. Third, association with expression levels provides a unique opportunity to identify the actual disease gene at the linkage or association locus. Fourth, individual or combined assessment of glutathione pathway genes that are regulated in the brain, may uncover additional neurodegenerative risk variants. Further establishment of *GSTO2 *and other glutathione metabolism genes in AD and PD awaits discovery and mechanistic studies of functional genetic variants.

## Methods

### Subjects and samples

#### LOAD and PD DNA samples

Unrelated subjects from six independent LOAD case-control series, consisting of Caucasians with an age-at-diagnosis (LOAD), evaluation (elderly controls) or death (autopsy series) ≥ 60 years, were utilized in this study (3,561 LOAD vs. 4,683 controls; Table 1 Table 2 and Table 3). Subjects with younger (60-80) and older (> 80) ages were assessed both separately and jointly, as per our prior reports[40-42] and given the age-specific effects observed for many LOAD risk variants, including APOE[43]. Four case-control series were comprised of Caucasian subjects collected in the United States, with three series collected at Mayo Clinic in Jacksonville, Florida (JS: 864 LOADs, 972 controls), Rochester, Minnesota, (RS: 597 LOADs, 2,406 controls) and an autopsy-confirmed series from the Brain Bank at Mayo Clinic Florida (AUT: 581 LOADs, 360 controls). The fourth Caucasian-American series was from the National Cell Repository for Alzheimer's Disease (NCRAD: 695 LOADs, 208 controls). These series were previously described in detail[40]. Two additional Caucasian series were from Poland[44] (PS: 479 LOADs, 186 controls) and Norway[45] (NW: 345 LOADs, 551 controls) were also included in this study. All clinical LOAD subjects had a diagnosis of probable or possible AD and all autopsied LOAD subjects of definite AD made according to NINCDS-ADRDA criteria[46]. All controls from the clinical Caucasian-American series had a clinical dementia rating score of 0. All autopsied LOAD brains had Braak scores of ≥ 4.0. Brains employed as controls had Braak scores of ≤ 2.5 but often had pathologies unrelated to AD.

We utilized age 80 as the arbitrary cutoff to define the older vs. younger LOAD series. This decision is partly based on the knowledge that the strongest genetic factor conferring LOAD risk, *APOE *ε4 has age-specific effects with highest effect sizes in younger ages (60-75)[47] (reviewed in [19]). Age-specific LOAD risk association has also been demonstrated for other genetic factors both by others[48,49] and by our group[50]. These results suggest that older vs. younger LOAD subjects may be heterogeneous and harbor different genetic risk factors. Consequently, we have divided our LOAD case-control series into older vs. younger age groups using the 80 year cutoff and analyze these series both separately and jointly in all of our studies assessing LOAD genetic risk, including the Mayo LOAD GWAS which was focused on the 60-80 year group[51].

Caucasian-American, unrelated PD patients and controls (PD: 678 PDs, 712 controls) were recruited and diagnosed as described[52,53], by a neurologist according to published criteria[54]. Control subjects lacked any history suggestive of parkinsonism. PDs with family history of parkinsonism (familial, PD FAM) and those without (sporadic, PD-SPO) were analyzed both separately and jointly. All DNA samples were isolated from peripheral blood, with the exception of samples in the autopsy series where DNA was isolated from donated brain tissue, as described in previous publications[40,44,45,52]. This study was approved by the appropriate institutional review board and appropriate informed consent was obtained from all participants.

#### RNA samples

Brain RNA for gene expression studies was obtained from the Mayo Clinic Autopsy (AUT) series, described above. These subjects were part of a larger expression GWAS (eGWAS) (in-press *Neurology *and *PloSGenetics*). AUT subjects with an age-at-death of 60-80 years were included in the Mayo LOAD GWAS[51]. RNA was extracted from the frozen cerebellum and temporal cortex samples of these autopsied subjects, where available, using the AB(Applied Biosystems) RNA was extracted from the frozen cerebellum and temporal cortex samples of these autopsied subjects, where available, using the Ambion RNAqueous kit according to the manufacturer’s instructions. The quantity and quality of the RNA samples were determined by the Agilent 2100 Bioanalyzer using the Agilent RNA 6000 Nano Chip. In total, 399 temporal cortex (202 LOADs, 197 Controls) and 374 cerebellar samples (197 LOADs, 177 Controls) were assessed.

### SNP genotyping

This study initially focused on four known coding variants from dbSNP within *GSTO1 *(rs4925, rs11509438) and *GSTO2 *(rs156697, rs34400162). We determined that rs11509438 was below the required minor allele frequency (MAF) cutoff of 5% (MAF = 3.3%) and rs34400162 was monomorphic in our series. We therefore focused on rs4925 and r156697 in all downstream analyses. Taqman (Applied Biosystems) was used to genotype rs4925 and rs155697 in all case-control series.

The genotypes for the eGWAS were obtained as part of the Mayo LOAD GWAS using Illumina (San Diego, CA) HumanHap300-Duo Genotyping BeadChips, and were analyzed with an Illumina BeadLab Station (Illumina, San Diego, CA), followed by quality control (QC), as previously described[51].

### Expression measurements

Transcript levels were measured using the Whole Genome DASL assay (Illumina, San Diego, CA). The RNA samples were randomized across the chips and plates using a stratified approach to ensure balance with respect to diagnosis, age, gender, RINs (RNA integrity numbers) and *APOE *genotype. Replicate samples were utilized for QC. Raw probe level mRNA expression data were exported from GenomeStudio software (Illumina Inc.) for preprocessing with background correction, variance stabilizing transformation, quantile normalization and probe filtering using the lumi package of BioConductor[55,56].

### Statistical analysis

#### Disease-risk association analysis

SNPs rs4925 and rs156697 were assessed for association with LOAD by multivariate logistic regression analysis using an allelic dosage model, adjusted for the following covariates: *APOE *ε4 dosage (0, 1, 2), age at diagnosis/evaluation/death, and gender. Analysis was executed for each of the six individual case control series and for the series combined, with a series covariate included in the model. All analyses were conducted separately on subjects of ages 60-80 years and those > 80 years, separately, and jointly.

Analysis for PD risk was done in a similar fashion. The PDs were classified as familial vs. sporadic based on the presence of family history of parkinsonism, in this cohort. We have therefore assessed the familial and sporadic PDs against the common PD control group both separately and jointly.

Meta analysis was also performed for rs4925 and rs156997 association with LOAD risk using the DerSimonian-Laird random effects model[57]. The younger (60-80) and older (> 80) age groups were analyzed separately, in addition to combined ages. Breslow-Day test for non-compatibility was used to test for series heterogeneity. Test statistics are reported for each series as well as the pooled test statistics from the random effects model.

#### Age-at-diagnosis association analysis

We employed age-at-diagnosis or death as the surrogate quantitative variable for age-at-onset in our LOAD subjects (3,561 LOADs). An additive model was assumed for the *GSTO *locus SNPs, with the minor allele dosage (0, 1, 2) as the independent variable, and APOE ε4 dosage (0, 1, 2), and gender as covariates. Analysis was executed for the LOADs both individually for the six series and with all series combined, where a series covariate was included in the model.

PD age-at-diagnosis analysis was done similarly, for the familial and sporadic PDs both individually and jointly. Both the disease risk and age-at-diagnosis associations for the two *GSTO *SNPs were conducted in StatsDirect (v.2.5.8).

#### Gene expression level association analysis

All *GSTO1 *and *GSTO2 *trancript measurements and *GSTO *locus *cis*SNP/transcript associations were conducted as part of our brain eGWAS[58]. Pathway analysis described below was also conducted using the *cis*SNP/transcript association results from this eGWAS. *GSTO *locus SNPs rs4925 and rs156697 were tested for association with brain levels of *GSTO1 *and *GSTO2 *transcripts measured in the cerebellum and temporal cortex of autopsied subjects as part of our eGWAS. Preprocessed probe transcript levels (*GSTO1 *= ILMN_2227573 and *GSTO2 *= ILMN_1740234) were used as the quantitative traits and the analyses were conducted as described (in-press *Neurology*[58], *PLoS Genetics*). An additive model was assumed, with the minor allele dosage (0, 1, 2) as the independent variable, and APOE ε4 dosage (0, 1, 2), age-at-death, gender, PCR plate, RIN, adjusted RIN2 (defined as (RIN-RINmean)2) as covariates. The cerebellar and temporal cortex results were analyzed separately. The autopsied LOADs and controls without AD pathology were analyzed both separately and jointly for rs4925 and rs156697, with the joint analysis including diagnosis as an additional covariate. Linear regression analysis to test for SNP/transcript associations were done with PLINK[59]. Box plots depicting transcript levels by SNP genotype were generated in R, for the residuals of the cerebellar and temporal cortex associations from the multivariate linear regression analysis described above.

Our brain eGWAS assessed association of brain transcript levels with their nearby *cis*SNPs described as those residing within the gene or its ± 100 kb flanking region. We determined that there were 20 additional *cis*SNPs tested for *GSTO1 *and *GSTO2 *in our eGWAS. The brain transcript level associations with these 20 *cis*SNPs were also assessed using the same analytical approach.

#### Linkage disequilibrium analysis

Linkage disequilibrium for the 20 *GSTO *locus *cis*SNPs, rs4925 and rs156697 was evaluated using the HapMap Caucasian (CEU) data[60] and assessed in HaploView version 4.1[61] with the solid spine algorithm.

#### Pathway analysis

In our brain eGWAS, we measured expression levels of 24,526 transcripts in 773 brain samples from the cerebellum and temporal cortex of autopsied subjects with Alzheimer's disease (AD, cerebellar n = 197, temporal cortex n = 202) and with other brain pathologies (control, cerebellar n = 177, temporal cortex n = 197) (in-press, *PLoS Genetics*). Association studies were carried out for the transcripts that could be detected in brain tissue, which is ~70% of all tested transcripts and for the 213,528 *cis*SNPs which reside within ± 100 kb of the genomic region of the transcript. We identified 686 genes with cerebellar transcript levels that are significantly influenced by *cis*SNPs in both the AD and control samples. Importantly, 625 of these genes could be tested in the temporal cortex, of which 471 were also significant for the transcript associations in this other brain region.

To discover the molecular pathways which harbor the top genes with significant *cis*SNP associations, we performed pathway analysis using MetaCore[33]. The total number of tested genes and the number of significant genes that belong to a MetaCore pathway were used to determine the molecular pathways that are significantly enriched for genes influenced by *cis*SNPs in the brain. Out of 11,897 tested genes with eGWAS data, 3,316 belong to at least one MetaCore pathway. Out of 686 genes with significant cerebellar transcript/*cis*SNP associations, 188 belong to at least one MetaCore pathway. Pathways with less than five tested genes were excluded from analysis. Fisher 2 × 2 test with mid-p value was calculated in R to determine significance of enrichment.

## Competing interests

N. Graff-Radford, M.D. has served as a consultant to Codman and received grant support from Elan Pharmaceutical Research, Pfizer Pharmaceuticals, Medivation, and Forrest. R.C. Petersen, M.D., Ph.D. has been a consultant to GE Healthcare and Elan Pharmaceuticals, has served on a data safety monitoring board in a clinical trial sponsored by Elan Pharmaceuticals, and a safety monitoring board for Wyeth Pharmaceuticals.

## Authors' contributions

MA carried out SNP genotyping assays, executed the disease risk and age at onset statistical analysis and helped to draft the manuscript. FZ participated in the organization and design of the gene expression study. HSC, CSY and AAN significantly contributed to the analysis of the gene expression data. RM participated in the collection of the gene expression data and carried out SNP genotyping assays. JEC and VSP significantly contributed to the design of the gene expression study and statistical analysis. MMC led the design, collection and analysis of SNP genotyping data used for the gene expression study. CNR and RP carried out SNP genotyping assays. TN, LM, KGM, GB and AIO provided technical expertise in the preparation, organization and maintenance of the RNA and DNA samples. SM, SM and CG participated in the analysis of the gene expression data. DS and FR participated in the collection of gene expression data. CPK and JJ supervised the collection of gene expression data. SBS, JOA, MB, RJU, ZKW, OAR, RCP, NRG and DWD collected and provided the case-control samples used in these studies. SGY significantly contributed to the conception and organization of the study. NET conceived of the study, and participated in its design and coordination and helped to draft the manuscript. All authors read and approved the final manuscript.

## Supplementary Material

Additional file 1 **"123011_GSTO_ms_SupplementaryText.docx", supplementary tables (SupplTable **1**, **2**), text and figure legends (SupplFigure **1**, **2a, b**), relevant to the manuscript**. Click here for file

Additional file 2 **"Suppl_Figure **1**_ExpressionPlot_120511_MA.tif" Supplementary Figure **1. Click here for file

Additional file 3 **"Suppl_Figure **2a**_Glutathione metabolism_Humanversion_12-02-2011.tif" Supplementary Figure **2a. Click here for file

Additional File 4 **"Suppl_Figure **2b**_MetaCoreLegend_pic.tif" Supplementary Figure **2b. Click here for file
