# Supplementary material for: Glutathione S-transferase omega genes in Alzheimer and Parkinson disease risk, age-at-diagnosis and brain gene expression: an association study with mechanistic implications
Source: Mol Neurodegener. 2012 Apr 11;7:13. doi: 10.1186/1750-1326-7-13 (PMC3393625; doi:10.1186/1750-1326-7-13)
Supplement: Additional file 1 — "123011_GSTO_ms_SupplementaryText.docx", supplementary tables (SupplTable 1, 2), text and figure legends (SupplFigure 1, 2a, b), relevant to the manuscript. [file 1750-1326-7-13-S1.DOC]

**Supplementary Table 1. Association of *GSTO* locus SNPs with LOAD and PD age-at-diagnosis:**

| a) LOAD age-at-diagnosis | | |  |  |  |  |
| --- | --- | --- | --- | --- | --- | --- |
| **rs#** | **Locus** | **Series** | **N (MAF)** | **Beta** | **SE** | **p-value** |
| rs156697 | GSTO2 | All | 3,490 (0.36) | 0.28 | 0.17 | 0.098 |
|  |  | JS | 853 (0.37) | -0.03 | 0.32 | NS |
|  |  | RS | 586 (0.35) | -0.05 | 0.44 | NS |
|  |  | AUT | 574 (0.38) | 0.56 | 0.52 | NS |
|  |  | NCRAD | 677 (0.35) | 0.31 | 0.37 | NS |
|  |  | NW | 341 (0.33) | 0.67 | 0.55 | NS |
|  |  | PS | 459 (0.37) | 0.20 | 0.33 | NS |
| rs4925 | GSTO1 | All | 3,489 (0.32) | 0.22 | 0.18 | NS |
|  |  | JS | 850 (0.32) | 0.68 | 0.52 | 0.195 |
|  |  | RS | 591 (0.31) | -0.17 | 0.45 | NS |
|  |  | AUT | 578 (0.33) | -0.27 | 0.34 | NS |
|  |  | NCRAD | 671 (0.31) | 0.38 | 0.38 | NS |
|  |  | NW | 331 (0.31) | 0.64 | 0.56 | NS |
|  |  | PS | 468 (0.35) | 0.15 | 0.35 | NS |
|  |  |  |  |  |  |  |
| b) PD age-at-diagnosis | | |  |  |  |  |
| **rs#** | **Locus** | **Series** | **N (MAF)** | **Beta** | **SE** | **p-value** |
| rs4925 | GSTO1 | PD-All | 667 (0.30) | 0.22 | 0.73 | NS |
|  |  | PD-SPO | 416 (0.33) | -0.02 | 0.91 | NS |
|  |  | PD FAM | 251 (0.27) | 0.06 | 1.21 | NS |
| rs156697 | GSTO1 | PD-All | 661 (0.35) | -0.06 | 0.70 | NS |
|  |  | PD-SPO | 411 (0.36) | -0.79 | 0.90 | NS |
|  |  | PD FAM | 250 (0.31) | 0.68 | 1.12 | NS |

**Supplementary Table 1. Association of *GSTO* locus SNPs with LOAD and PD age-at-diagnosis:** Results of multivariate linear regression analysis are shown for each SNP, each series individually and for the combined series. N=number of subjects, MAF=minor allele frequency, Beta=coefficient of variation for the SNP additive genotype variable; SE=standard error; NS=not significant. For LOAD series, all age groups were assessed jointly.

**Supplementary Table 2: Association of all *GSTO* locus *cis*SNPs with brain *GSTO2* expression levels**

**Supplementary Table 2: Association of all *GSTO* locus *cis*SNPs with brain *GSTO2* expression levels:** Results of multivariate linear regression analysis testing association of all *cis*SNPs at the *GSTO* locus with cerebellar (Cer) and temporal cortex (Tx) levels of *GSTO2* in the combined autopsied subjects with and without AD pathology. Diagnosis was used as a covariate in all analyses, in addition to the others discussed in Methods. Beta coefficient and p value of association between the transcript levels and the *cis*SNP are shown for each analyzed brain region. SNP Haploview # is the number representing the SNP on the LD figure (Supplementary Fig. 1).

**Supplementary Figure Legends:**

**Supplementary Figure 1: Data plots of all *GSTO* locus *cis*SNPs tested for association with brain expression levels of *GSTO2*:** P-values shown on the y-axis were transformed using –log10 and are plotted against the position (Kbp) of each *cis*SNP along the chromosome, which is shown on the x-axis. The SNPs are numbered 1-22 and correspond to the SNP Haploview# in Supplementary Table 2. Transformed p values for the *cis*SNP associations with *GSTO2* expression levels in the cerebellum (CER) are shown by the blue lines with each *cis*SNP p-value depicted as a diamond; and those for the temporal cortex (TCX) are shown in pink with squares. The relative locations of *GSTO1* (1) and *GSTO2* (2) are shown as red and green horizontal arrows respectively. The SNPs rs4925 and rs156697 are likewise highlighted by red and green boxes respectively. The LD across the locus is represented by a plot generated with Haploview, using data from the HapMap Caucasian series and solid spine algorithm. The numbers on the LD plot are the D’ values.

**Supplementary Figure 2: Glutathione molecular pathway:** The 686 genes with significant *cis*SNP associations in the cerebellum were analyzed by MetaCore to identify pathways that are enriched in genes with significant regulation in the brain. a) The significant glutathione metabolism pathway. The red stars denote the genes tested in our eGWAS and found to have significant *cis*SNPs. The blue stars denote those that are tested, but without significant *cis*SNPs. Two of the non-significant tested genes, *GCLM* and *GGT7*, are not represented on the MetaCore map. b) MetaCore legends used in Supplementary Figure 2a.

**Supplementary Text:**

**Glutathione metabolism pathway description from MetaCore:**

Glutathione can be found in the cell in oxidized (**Glutathione disulfide**) and reduced (**Glutathione**) form. Reduced glutathione can be either directly formed from **Glutathione disulfide** as the result of activity of Glutathione reductase (**GSHR**) , or from conjugation of **L-Cysteinyl-glycine** with **(L)-Glutamic acid** catalyzed by Gamma-glutamyltranspeptidase 1 precursor (**GGT1**) or by Gamma-glutamyltransferase light chain 2 (**GGTL3**) .

**L-Cysteinyl-glycine** is formed as a result of glutathione conjugation to the **L-Amino acid** moiety catalyzed by **GGT1** and **GGTL3**. This reaction results in formation of **Gamma-(L)-glutamyl-aminoacid** which is converted by Gamma-glutamylcyclotransferase (**GCTG**) to the **5-Oxo-(L)-proline** and **L-Amino acid**. **5-Oxo-(L)-proline** is then reduced to **(L)-Glutamic acid** by 5-Oxoprolinase (**OPLA**) , **(L)-Glutamic acid** also produced in the **Glutathione** degradation during the **R-S-Alanylglycine** formation step.

**5-Oxo-(L)-proline** and **(L)-Cysteine** are produced as the result of **Gamma-(L)-glutamyl-(L)-cysteine** cleavage catalyzed by **GCTG**. **Gamma-(L)-glutamyl-(L)-cysteine** itself is synthesized by Glutamate-cysteine ligase catalytic subunit (**GCL cat**) from **(L)-Glutamic acid** and from **(L)-Cysteine**. Glutathione synthetase (**GSHB**) catalyzes subsequent conjugation of **Gamma-(L)-glutamyl-(L)-cysteine** and **Glycine** to form **Glutathione**. **Glycine** is supplied from **L-Cysteinyl-glycine** degradation to **Glycine** and to **(L)-Cysteine**, as well as from the **R-S-Alanylglycine** degradation step.

**Glutathione** (reduced) reacts with various substrates (**RX**). Those reactions are carried out by a set of enzymes: Glutathione S-transferase A1 (**GSTA1**), Glutathione S-transferase A2 (**GSTA2**), Glutathione S-transferase A3 (**GSTA3**), Glutathione S-transferase A4 (**GSTA4**), Glutathione S-transferase A5 (**GSTA5**), Glutathione S-transferase M1 (**GSTM1**), Glutathione S-transferase M2 (**GSTM2**), Glutathione S-transferase M3 (**GSTM3**), Glutathione S-transferase M4 (**GSTM4**), glutathione S-transferase M5 (**GSTM5**), Glutathione transferase zeta 1 (maleylacetoacetate isomerase) (**MAAI**) , Glutathione transferase omega-1 (**GSTO1**), Glutathione transferase omega-2 (**GSTO2**), Glutathione S-transferase theta-1 (**GSTT1**), Glutathione S-transferase theta-2 (**GSTT2**), Glutathione S-transferase kappa 1 (**GSTK1**) , **Glutathione S-transferase pi 1** (**GSTP1**), Microsomal glutathione S-transferase 1 (**MGST**), Microsomal glutathione S-transferase 2 (**MGST2**), and Microsomal glutathione S-transferase 3 (**MGST3**) . These reactions produce **R-S-Glutathione** (glutathione conjugated to a moiety) products. Subsequently **R-S-Glutathione** lose their **(L)-Glutamic acid** moieties in reactions catalyzed by **GGT1** and **GGTL3** to produce **R-S-Alanylglycine** that is further degraded to **R-S-Alanine** by Aminopeptidase N (**CD13**).

**Glutathione disulfide** can be formed directly from **Glutathione** in the reaction catalyzed by Glutathione peroxidase 4 (**GPX4 (PHGPx)**), Glutathione peroxidase 1 (**GPX1**), Glutathione peroxidase 2 (**GPX2**), Glutathione peroxidase 3 (plasma) (**GPX3**), Glutathione peroxidase 6 (**GPX6**), Glutathione peroxidase 7 (**GPX7**), Glutathione peroxidase 5 (**GPX5**) and **GSHR**. **Glutathione disulfide** can also be formed through ascorbate metabolism.

**Other Supplementary References:**

1. Barrett JC, Fry B, Maller J, Daly MJ: **Haploview: analysis and visualization of LD and haplotype maps.** *Bioinformatics* 2005, **21:**263-265.

2. Frazer KA, Ballinger DG, Cox DR, Hinds DA, Stuve LL, Gibbs RA, Belmont JW, Boudreau A, Hardenbol P, Leal SM, et al: **A second generation human haplotype map of over 3.1 million SNPs.** *Nature* 2007, **449:**851-861.

3. Ekins S, Nikolsky Y, Bugrim A, Kirillov E, Nikolskaya T: **Pathway mapping tools for analysis of high content data.** *Methods Mol Biol* 2007, **356:**319-350.

4. Barker JE, Heales SJ, Cassidy A, Bolanos JP, Land JM, Clark JB: **Depletion of brain glutathione results in a decrease of glutathione reductase activity; an enzyme susceptible to oxidative damage.** *Brain Res* 1996, **716:**118-122.

5. Dickinson DA, Forman HJ: **Glutathione in defense and signaling: lessons from a small thiol.** *Ann N Y Acad Sci* 2002, **973:**488-504.

6. Leh H, Courtay C, Gerardin P, Wellman M, Siest G, Visvikis A: **Cloning and expression of a novel type (III) of human gamma-glutamyltransferase truncated mRNA.** *FEBS Lett* 1996, **394:**258-262.

7. Heinle H, Wendel A: **[Does a modified gamma-glutamyl cycle exist in human erythrocytes (author's transl)].** *Hoppe Seylers Z Physiol Chem* 1976, **357:**1459-1463.

8. Fink ML, Chung SI, Folk JE: **gamma-Glutamylamine cyclotransferase: specificity toward epsilon-(L-gamma-glutamyl)-L-lysine and related compounds.** *Proc Natl Acad Sci U S A* 1980, **77:**4564-4568.

9. Danson JW, Trawick ML, Cooper AJ: **Spectrophotometric assays for L-lysine alpha-oxidase and gamma-glutamylamine cyclotransferase.** *Anal Biochem* 2002, **303:**120-130.

10. Srivenugopal KS, Ali-Osman F: **Activity and distribution of the cysteine prodrug activating enzyme, 5-oxo-L-prolinase, in human normal and tumor tissues.** *Cancer Lett* 1997, **117:**105-111.

11. Chen X, Schecter RL, Griffith OW, Hayward MA, Alpert LC, Batist G: **Characterization of 5-oxo-L-prolinase in normal and tumor tissues of humans and rats: a potential new target for biochemical modulation of glutathione.** *Clin Cancer Res* 1998, **4:**131-138.

12. Weber P, Jager M, Bangsow T, Knell G, Piechaczek K, Koch J, Wolf S: **Kinetic parameters and tissue distribution of 5-oxo-L-prolinase determined by a fluorimetric assay.** *J Biochem Biophys Methods* 1999, **38:**71-82.

13. Sriram R, Ali-Osman F: **Purification and biochemical characterization of gamma-glutamylcysteine synthetase from a human malignant astrocytoma cell line.** *Biochem Mol Biol Int* 1993, **30:**1053-1060.

14. Misra I, Griffith OW: **Expression and purification of human gamma-glutamylcysteine synthetase.** *Protein Expr Purif* 1998, **13:**268-276.

15. Beutler E, Gelbart T: **Improved assay of the enzymes of glutathione synthesis: gamma-glutamylcysteine synthetase and glutathione synthetase.** *Clin Chim Acta* 1986, **158:**115-123.

16. Ristoff E, Hebert C, Njalsson R, Norgren S, Rooyackers O, Larsson A: **Glutathione synthetase deficiency: is gamma-glutamylcysteine accumulation a way to cope with oxidative stress in cells with insufficient levels of glutathione?** *J Inherit Metab Dis* 2002, **25:**577-584.

17. Stenberg G, Bjornestedt R, Mannervik B: **Heterologous expression of recombinant human glutathione transferase A1-1 from a hepatoma cell line.** *Protein Expr Purif* 1992, **3:**80-84.

18. Ahmad H, Singhal SS, Saxena M, Awasthi YC: **Characterization of two novel subunits of the alpha-class glutathione S-transferases of human liver.** *Biochim Biophys Acta* 1993, **1161:**333-336.

19. Pulford DJ, Hayes JD: **Characterization of the rat glutathione S-transferase Yc2 subunit gene, GSTA5: identification of a putative antioxidant-responsive element in the 5'-flanking region of rat GSTA5 that may mediate chemoprotection against aflatoxin B1.** *Biochem J* 1996, **318 ( Pt 1):**75-84.

20. Hubatsch I, Ridderstrom M, Mannervik B: **Human glutathione transferase A4-4: an alpha class enzyme with high catalytic efficiency in the conjugation of 4-hydroxynonenal and other genotoxic products of lipid peroxidation.** *Biochem J* 1998, **330 ( Pt 1):**175-179.

21. Johansson AS, Mannervik B: **Human glutathione transferase A3-3, a highly efficient catalyst of double-bond isomerization in the biosynthetic pathway of steroid hormones.** *J Biol Chem* 2001, **276:**33061-33065.

22. Tetlow N, Liu D, Board P: **Polymorphism of human Alpha class glutathione transferases.** *Pharmacogenetics* 2001, **11:**609-617.

23. Vorachek WR, Pearson WR, Rule GS: **Cloning, expression, and characterization of a class-mu glutathione transferase from human muscle, the product of the GST4 locus.** *Proc Natl Acad Sci U S A* 1991, **88:**4443-4447.

24. Ross VL, Board PG: **Molecular cloning and heterologous expression of an alternatively spliced human Mu class glutathione S-transferase transcript.** *Biochem J* 1993, **294 ( Pt 2):**373-380.

25. Klone A, Hussnatter R, Sies H: **Cloning, sequencing and characterization of the human alpha glutathione S-transferase gene corresponding to the cDNA clone pGTH2.** *Biochem J* 1992, **285 ( Pt 3):**925-928.

26. Tsuchida S, Maki T, Sato K: **Purification and characterization of glutathione transferases with an activity toward nitroglycerin from human aorta and heart. Multiplicity of the human class Mu forms.** *J Biol Chem* 1990, **265:**7150-7157.

27. Alin P, Mannervik B, Jornvall H: **Structural evidence for three different types of glutathione transferase in human tissues.** *FEBS Lett* 1985, **182:**319-322.

28. Zhong S, Spurr NK, Hayes JD, Wolf CR: **Deduced amino acid sequence, gene structure and chromosomal location of a novel human class Mu glutathione S-transferase, GSTM4.** *Biochem J* 1993, **291 ( Pt 1):**41-50.

29. Takahashi Y, Campbell EA, Hirata Y, Takayama T, Listowsky I: **A basis for differentiating among the multiple human Mu-glutathione S-transferases and molecular cloning of brain GSTM5.** *J Biol Chem* 1993, **268:**8893-8898.

30. Jakobsson PJ, Mancini JA, Ford-Hutchinson AW: **Identification and characterization of a novel human microsomal glutathione S-transferase with leukotriene C4 synthase activity and significant sequence identity to 5-lipoxygenase-activating protein and leukotriene C4 synthase.** *J Biol Chem* 1996, **271:**22203-22210.

31. Tong Z, Board PG, Anders MW: **Glutathione transferase zeta catalyses the oxygenation of the carcinogen dichloroacetic acid to glyoxylic acid.** *Biochem J* 1998, **331 ( Pt 2):**371-374.

32. Polekhina G, Board PG, Blackburn AC, Parker MW: **Crystal structure of maleylacetoacetate isomerase/glutathione transferase zeta reveals the molecular basis for its remarkable catalytic promiscuity.** *Biochemistry* 2001, **40:**1567-1576.

33. Board PG, Coggan M, Chelvanayagam G, Easteal S, Jermiin LS, Schulte GK, Danley DE, Hoth LR, Griffor MC, Kamath AV, et al: **Identification, characterization, and crystal structure of the Omega class glutathione transferases.** *J Biol Chem* 2000, **275:**24798-24806.

34. Whitbread AK, Tetlow N, Eyre HJ, Sutherland GR, Board PG: **Characterization of the human Omega class glutathione transferase genes and associated polymorphisms.** *Pharmacogenetics* 2003, **13:**131-144.

35. Mainwaring GW, Williams SM, Foster JR, Tugwood J, Green T: **The distribution of theta-class glutathione S-transferases in the liver and lung of mouse, rat and human.** *Biochem J* 1996, **318 ( Pt 1):**297-303.

36. Rossjohn J, McKinstry WJ, Oakley AJ, Verger D, Flanagan J, Chelvanayagam G, Tan KL, Board PG, Parker MW: **Human theta class glutathione transferase: the crystal structure reveals a sulfate-binding pocket within a buried active site.** *Structure* 1998, **6:**309-322.

37. Morel F, Rauch C, Petit E, Piton A, Theret N, Coles B, Guillouzo A: **Gene and protein characterization of the human glutathione S-transferase kappa and evidence for a peroxisomal localization.** *J Biol Chem* 2004, **279:**16246-16253.

38. Li J, Xia Z, Ding J: **Thioredoxin-like domain of human kappa class glutathione transferase reveals sequence homology and structure similarity to the theta class enzyme.** *Protein Sci* 2005, **14:**2361-2369.

39. Singh SV, Ahmad H, Kurosky A, Awasthi YC: **Purification and characterization of unique glutathione S-transferases from human muscle.** *Arch Biochem Biophys* 1988, **264:**13-22.

40. Ali-Osman F, Akande O, Antoun G, Mao JX, Buolamwini J: **Molecular cloning, characterization, and expression in Escherichia coli of full-length cDNAs of three human glutathione S-transferase Pi gene variants. Evidence for differential catalytic activity of the encoded proteins.** *J Biol Chem* 1997, **272:**10004-10012.

41. DeJong JL, Morgenstern R, Jornvall H, DePierre JW, Tu CP: **Gene expression of rat and human microsomal glutathione S-transferases.** *J Biol Chem* 1988, **263:**8430-8436.

42. Lin MT, Beal MF: **Mitochondrial dysfunction and oxidative stress in neurodegenerative diseases.** *Nature* 2006, **443:**787-795.

43. White AR, Collins SJ, Maher F, Jobling MF, Stewart LR, Thyer JM, Beyreuther K, Masters CL, Cappai R: **Prion protein-deficient neurons reveal lower glutathione reductase activity and increased susceptibility to hydrogen peroxide toxicity.** *Am J Pathol* 1999, **155:**1723-1730.

44. Tam NN, Gao Y, Leung YK, Ho SM: **Androgenic regulation of oxidative stress in the rat prostate: involvement of NAD(P)H oxidases and antioxidant defense machinery during prostatic involution and regrowth.** *Am J Pathol* 2003, **163:**2513-2522.

45. Maiorino M, Chu FF, Ursini F, Davies KJ, Doroshow JH, Esworthy RS: **Phospholipid hydroperoxide glutathione peroxidase is the 18-kDa selenoprotein expressed in human tumor cell lines.** *J Biol Chem* 1991, **266:**7728-7732.

46. Chu FF, Doroshow JH, Esworthy RS: **Expression, characterization, and tissue distribution of a new cellular selenium-dependent glutathione peroxidase, GSHPx-GI.** *J Biol Chem* 1993, **268:**2571-2576.

47. Chu FF, Esworthy RS, Doroshow JH, Doan K, Liu XF: **Expression of plasma glutathione peroxidase in human liver in addition to kidney, heart, lung, and breast in humans and rodents.** *Blood* 1992, **79:**3233-3238.

48. Esworthy RS, Chu FF, Paxton RJ, Akman S, Doroshow JH: **Characterization and partial amino acid sequence of human plasma glutathione peroxidase.** *Arch Biochem Biophys* 1991, **286:**330-336.

49. Kryukov GV, Castellano S, Novoselov SV, Lobanov AV, Zehtab O, Guigo R, Gladyshev VN: **Characterization of mammalian selenoproteomes.** *Science* 2003, **300:**1439-1443.

50. De Vega L, Perez Fernandez R, Martin Mateo MC, Bustamante J, Bustamante A, Herrero AM, Bustamante Munguira E: **Study of the activity of glutathione-peroxidase, glutathione-transferase, and glutathione-reductase in renal transplants.** *Transplant Proc* 2003, **35:**1346-1350.

51. Hall L, Williams K, Perry AC, Frayne J, Jury JA: **The majority of human glutathione peroxidase type 5 (GPX5) transcripts are incorrectly spliced: implications for the role of GPX5 in the male reproductive tract.** *Biochem J* 1998, **333 ( Pt 1):**5-9.
