# Supplementary figures and images for: Glutathione S-transferase omega genes in Alzheimer and Parkinson disease risk, age-at-diagnosis and brain gene expression: an association study with mechanistic implications
Source: Mol Neurodegener. 2012 Apr 11;7:13. doi: 10.1186/1750-1326-7-13 (PMC3393625; doi:10.1186/1750-1326-7-13)

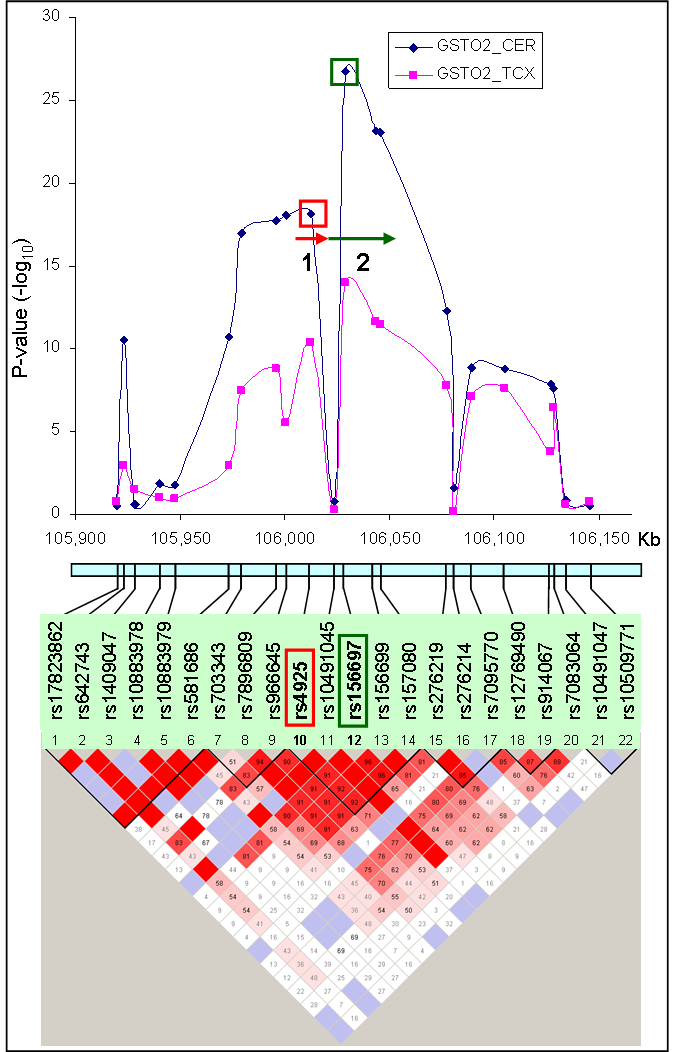

Supplement: Additional file 2 — "Suppl_Figure 1_ExpressionPlot_120511_MA.tif" Supplementary Figure 1. [file 1750-1326-7-13-S2.TIFF]

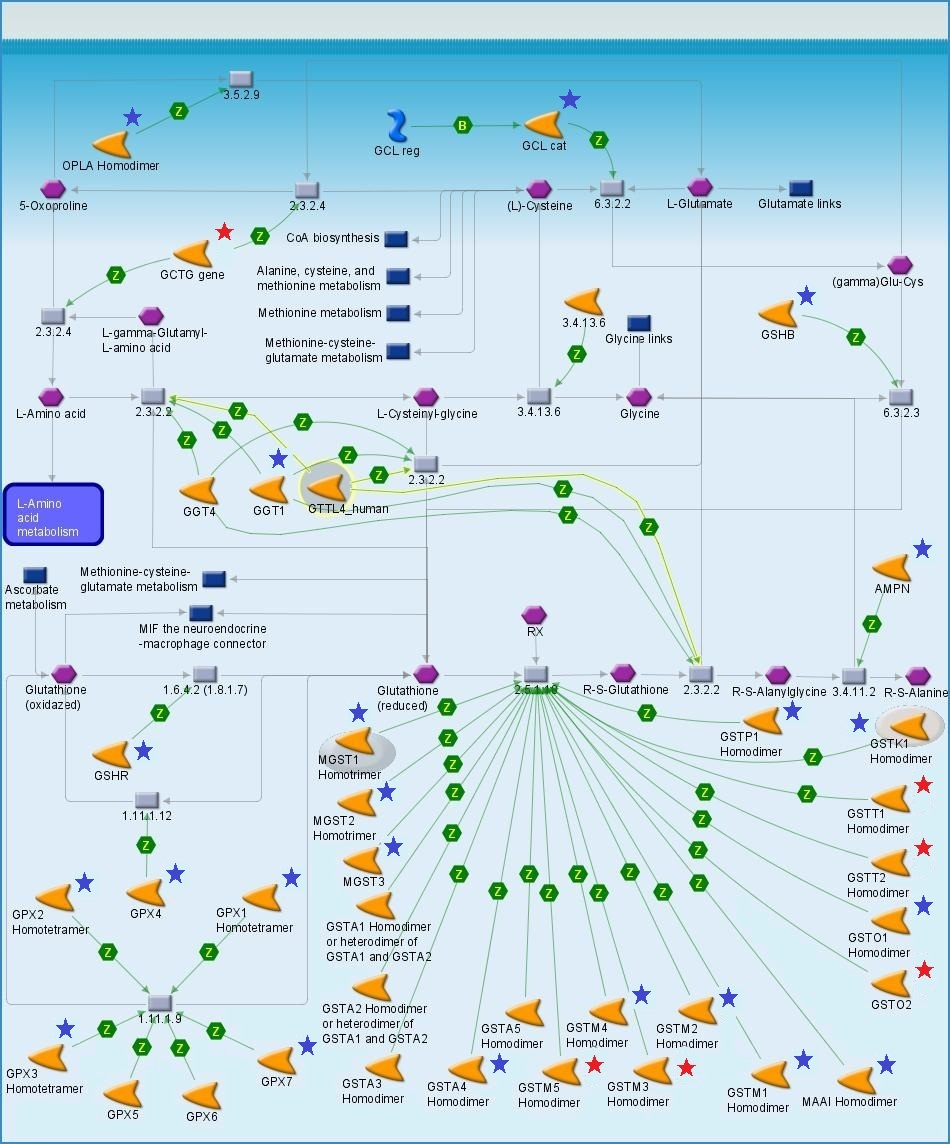

Supplement: Additional file 3 — "Suppl_Figure 2a_Glutathione metabolism_Humanversion_12-02-2011.tif" Supplementary Figure 2a. [file 1750-1326-7-13-S3.TIFF]

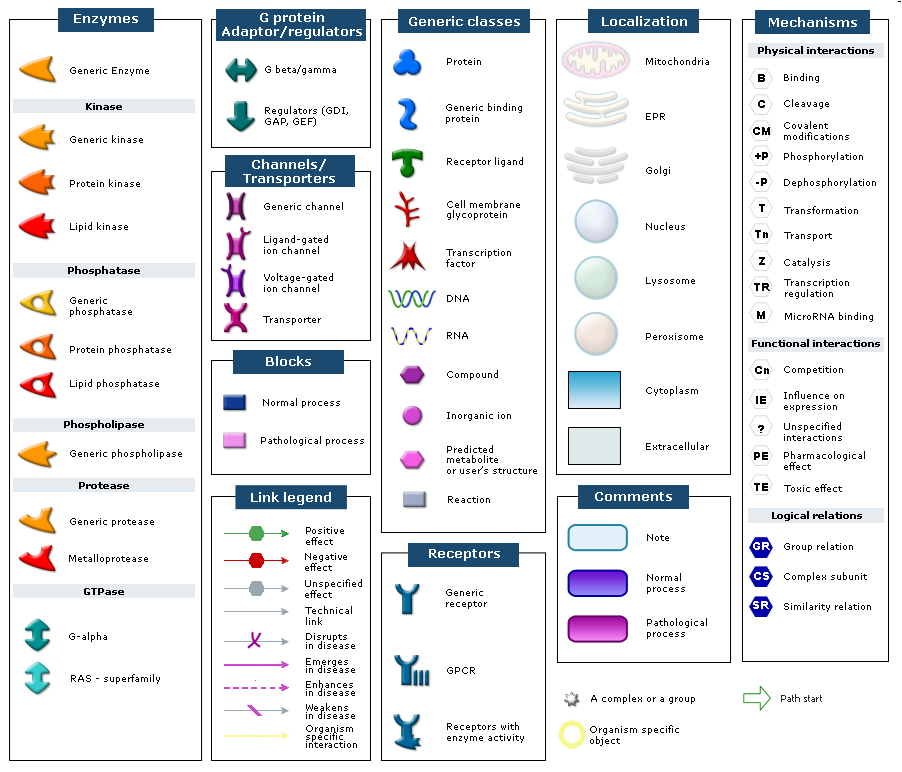

Supplement: Additional File 4 — "Suppl_Figure 2b_MetaCoreLegend_pic.tif" Supplementary Figure 2b. [file 1750-1326-7-13-S4.TIFF]
